# 4046 Museum and Arts-Space Programming Intended to Improve Health: Interim Survey Results

**DOI:** 10.1017/cts.2020.276

**Published:** 2020-07-29

**Authors:** Ian Koebner, Helen Chatterjee, Daniel J. Tancredi, Claudia M. Witt, Pier Luigi Sacco, Ruchi Rawal, Fred J Meyers

**Affiliations:** 1 University of California, Davis; 2 University College London; 3 University of Zurich; 4 IULM University

## Abstract

OBJECTIVES/GOALS: Many museums and art spaces conduct programming intended to improve health outcomes, but arts professionals’ perceptions of these programs are not well known. This study describes arts professionals’ experiences with museum and art-space interventions intended to improve health. METHODS/STUDY POPULATION: A 14-item digital Qualtrics survey was administered to museums and arts organizations selected using snowball sampling. The survey was sent to a range of arts and cultural organizations and professional membership bodies in the US and UK. Survey questions assessed the range of audiences involved in health programs, what types of activities museums and arts organizations are offering to support health outcomes, and how programs are evaluated. RESULTS/ANTICIPATED RESULTS: From 10/30/19-11/19/19, 151 surveys were completed; 66 respondents (44%) have a museum/arts in health program. Common target populations include individuals with mental health concerns (33, 22%) and older adults (26, 17%). Improving wellbeing (56, 37%) and social isolation (50, 33%) were the most common intended outcomes. Respondents reported using a variety of program evaluation methods including formal (23, 15%), informal (31, 21%), and anecdotal (37, 25%). Interviews are planned with a purposive sample of respondents conducting, or interested in conducting, a program for individuals with chronic pain and those formally evaluating their programs. DISCUSSION/SIGNIFICANCE OF IMPACT: Interim survey responses indicate many cultural organizations engage in programming intended to improve health outcomes. Understanding the cultural sector’s current efforts to improve health represents an initial step in translating these efforts into effective intersectoral research partnerships.

